# SWATH-MS data of *Drosophila melanogaster* proteome dynamics during embryogenesis

**DOI:** 10.1016/j.dib.2016.10.009

**Published:** 2016-10-24

**Authors:** Bertrand Fabre, Dagmara Korona, Daniel J.H. Nightingale, Steven Russell, Kathryn S. Lilley

**Affiliations:** a Cambridge Centre for Proteomics, Department of Biochemistry, University of Cambridge, Cambridge, U.K; b Department of Biochemistry, University of Cambridge, Cambridge, U.K; c Department of Genetics, University of Cambridge, Cambridge, U.K; d Cambridge Systems Biology Centre, University of Cambridge, Cambridge, U.K

**Keywords:** Early embryo development, Mass-spectrometry, SWATH

## Abstract

Embryogenesis is one of the most important processes in the life of an animal. During this dynamic process, progressive cell division and cellular differentiation are accompanied by significant changes in protein expression at the level of the proteome. However, very few studies to date have described the dynamics of the proteome during the early development of an embryo in any organism. In this dataset, we monitor changes in protein expression across a timecourse of more than 20 h of *Drosophila melanogaster* embryonic development. Mass-spectrometry data were produced using a SWATH acquisition mode on a Sciex Triple-TOF 6600. A spectral library built in-house was used to analyse these data and more than 1950 proteins were quantified at each embryonic timepoint. The files presented here are a permanent digital map and can be reanalysed to test against new hypotheses. The data have been deposited with the ProteomeXchange Consortium with the dataset identifier PRIDE: PXD0031078.

**Specifications Table**

**Table:** 

Subject area	*Biology.*
More specific subject area	*Proteomics, Drosophila melanogaster, Embryogenesis.*
Type of data	*Table, figure.*
How data was acquired	*Mass spectrometry, SWATH acquisition mode on a Sciex Triple-TOF 6600.*
Data format	*Analysed.*
Experimental factors	*SWATH MS analysis of different embryo stages.*
Experimental features	*Different developmental stages of Drosophila melanogaster embryos were collected, lysed, digested with trypsin and analysed in SWATH acquisition mode on a Sciex Triple-TOF 6600. The data were analysed using Spectronaut*^*TM*^.
Data source location	*Cambridge, United Kingdom.*
Data accessibility	*SWATH data have been deposited to the ProteomeXchange Consortium via the PRIDE partner repository with the dataset identifier PRIDE:*PXD0031078.

**Value of the data**

• First MS data available for a time course covering more than 20 h of embryo development.
• More than 1950 proteins identified (FDR<0.001%) and quantified at every timepoint using a spectral library built in-house.
• SWATH permanent digital maps that can be searched with new hypotheses (using optimized spectral library, analysis of post–translational modifications, etc.).

## 1 Data

**Fig. 1:**
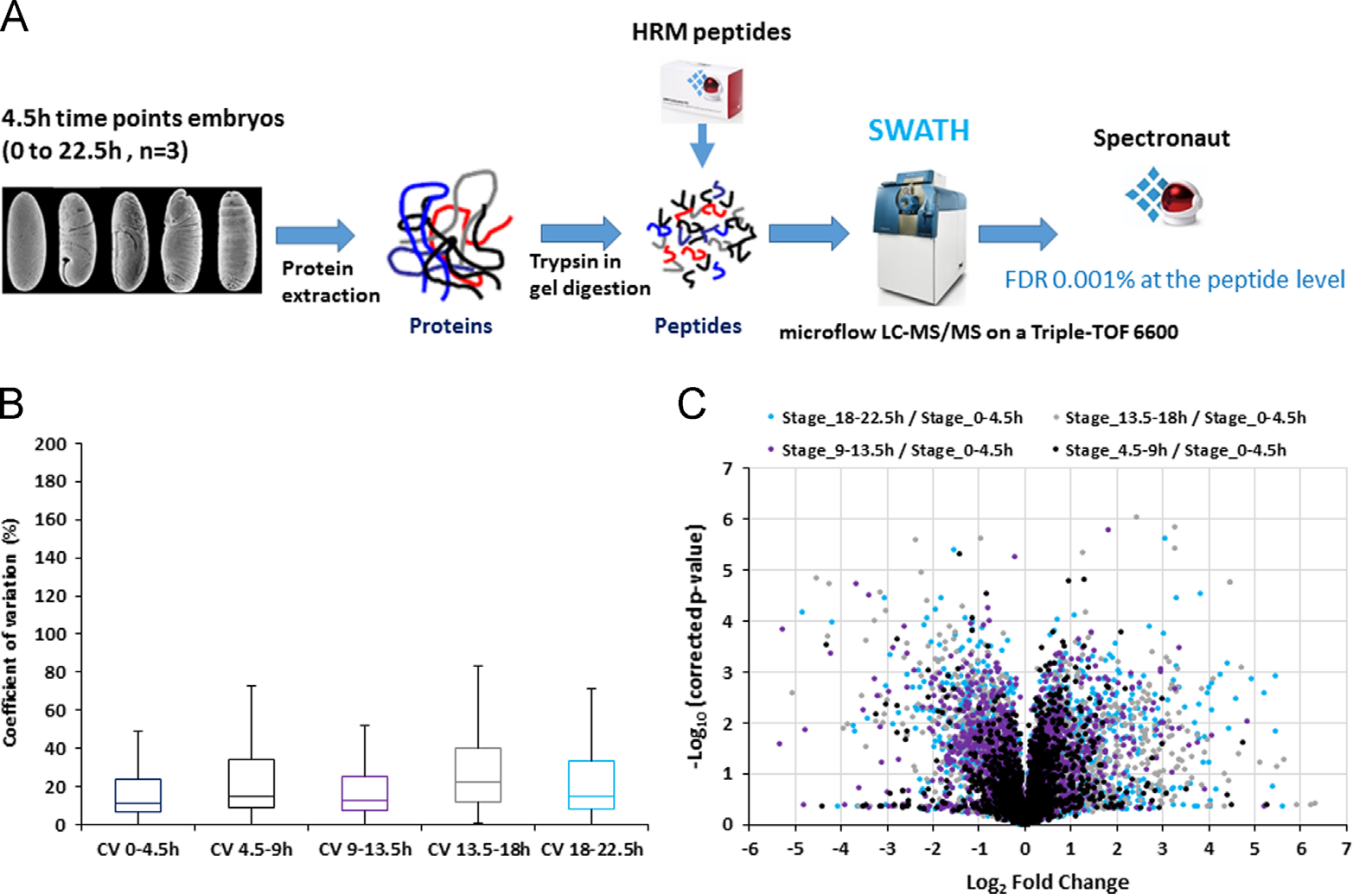
A) Overall strategy to measure changes in protein expression levels across *D. melanogaster* embryonic development by SWATH-MS. Embryos were collected at five 4.5 h timepoints to provide a developmental timecourse. Proteins were extracted, digested with trypsin and HRM peptides were spiked into the samples before injection. The samples were analysed using a SWATH acquisition mode on a Sciex Triple-TOF 6600. The resulting files were analysed with MaxQuant and Spectronaut^TM^. B) The Coefficient of variation (CV) between the biological replicates were calculated for each protein. CVs values were analysed using box plots with Excel. C) Volcano-plot representing the Benjamini–Hochberg corrected *p*-value as a function of the Log_2_ Fold Change for each protein between each timepoint and the 0–4.5 h timepoint.

The protein expression in *Drosophila melanogaster* embryos was monitored by SWATH-MS [1]. Proteins were extracted from a total lysate of embryos using a buffer containing 4% SDS. Samples were prepared using a GeLCMS preparation method and proteins were digested with trypsin. The peptides were analysed using the SWATH acquisition mode on a Sciex TripleTOF 6600. The workflow used in this study is illustrated in Fig. 1A. The dataset presented here is associated with prior publication [2] and includes all the SWATH raw files and output files from the Spectronaut^TM^ analysis (Supplementary Table).

## 2 Experimental design, materials and methods

### 2.1 Fly Lines, embryo collection and protein extraction

*D. melanogaster* sequenced iso-1 strain flies from the Bloomington Stock Centre (*y*^*1*^*; Gr22b*^*iso-1*^ *Gr22d*^*iso-1*^ *cn*^*1*^ *CG33964*^*iso-1*^ *bw*^*1*^ *sp*^*1*^*; LysC*^*iso-1*^ *MstProx*^*iso-1*^ *GstD5*_*iso-1*_ *Rh6*^*1*^) were kept in a 12-h light-dark cycle at 25 °C and 75% relative humidity on standard yeast-cornmeal media. Embryos were collected every 4.5 h after egg laying and aged to generate a developmental time course (0–4.5 h, 4.5–9 h, 9–13.5 h, 13.5–18 h and 18–22.5 h). Embryos were then dechorionated with 50% bleach, washed with water, frozen in liquid nitrogen and kept at −80 °C. Three independent biological replicates were collected for each time point. Embryos samples were lysed with a Dounce homogenizer (50 strokes per sample) in a buffer containing Tris 50 mM pH 7.5, 4% SDS, protease inhibitor (Complete, Roche). The samples were boiled for 5 min at 95 °C, sonicated (Bioruptor (Diagenode), position high, 30 s on, 30 s off for 10 min), centrifuged at 14,000 g for 10 min and the pellets discarded.

### 2.2 Sample preparation for mass-spectrometry analysis

In gel digestion was used as the sample preparation method as described in [3] with the following modifications. Protein concentration was measured using the detergent compatible (DC) protein assay (Bio-Rad). Loading buffer (Tris 40 mM pH 7.5, 2% SDS, 10% glycerol, 25 mM DTT final concentration) was added to the samples and they were boiled for 5 min at 95 °C. Iodoacetamide at a final concentration of 60 mM was used to alkylate cysteines for 30 min at room temperature in the dark. 100 µg of protein per condition were loaded on a SDS-PAGE gel (acrylamide concentration of 4% for stacking and 12% for resolving). Proteins were concentrated in one band in the resolving gel (approximately 1 cm long). Gels were stained with colloidal coomassie, cut into 1 mm^3^ pieces and washed several times with a 50% acetonitrile (ACN), 25 mM ammonium bicarbonate pH 8.0 (AB) solution. Gel bands were dried with ACN and swollen with trypsin (Promega) in 50 mM AB. 2 µg of trypsin was used for protein digestion overnight at 37 °C. The resulting peptides were extracted from the gel by two consecutive incubations in 10% formic acid, acetonitrile (1:1) for 15 min at 37 °C. The extractions were pooled with the initial digestion supernatant for each sample, dried in a Speed-Vac and resuspended in 100 µl of 3% ACN, 0.1% formic acid for mass-spectrometry analysis. HRM peptides (Biognosys) were added to each sample before injection into the mass spectrometer.

### 2.3 Generation of the spectral library

For the generation of the SWATH assay library, a high pH reverse phase fractionation of 1 mg of a protein sample from embryos collected over a 24 h period was performed. Peptides were loaded onto an Acquity bridged ethyl hybrid C18 UPLC column (Waters, 2.1 mm inner diameter 150 mm, 1.7 µm particle size), and separated with a gradient from 0 to 35% buffer B in 50 min and 35 to 100% buffer B in 7 min (buffer A: 20 mM ammonium formate pH10; buffer B: 20 mM ammonium formate pH10 / 80% ACN) at a flow rate of 0.244 ml min^−1^. Chromatographic performance was monitored by sampling the eluate with a diode array detector (Acquity UPLC, Waters) scanning wavelengths from 210 to 400 nm. Fractions were collected at 1 min intervals. Thirty-five fractions were collected, dried with a Speed-Vac and resuspended in 3% acetonitrile / 0.1% formic acid. Thirty-two fractions were analysed in Information Dependent Acquisition (IDA) mode on a TripleTOF 6600 mass spectrometer (Sciex). Half of the peptides from each fraction were injected. To each sample, HRM peptides (Biognosys) were added before analysis. Mass spectrometry analyses were performed on a TripleTOF 6600 mass spectrometer equipped with a Duospray ion source (Sciex) and coupled to an ACQUITY UPLC System (Waters). The samples were injected onto a MicroLC column (150 mm longx0.3 mm inner diameter) with ChromXP C18CL, 300 Å pore size, 3 μm diameter particles (Sciex). Samples were run with a 49 min gradient from 3–40% solvent B (solvent A 0.1% formic acid, 5% DMSO in water; solvent B: 0.1% formic acid, 5% DMSO in acetonitrile) at a flow rate of 5 µl/min. Data were acquired using an ion spray voltage of 5.5 kV, curtain gas at 25 psi and nebulizer gas at 10 psi. An IDA method was used with a MS survey range set between 350 and 1250 m/z (250 ms accumulation time) followed by MS/MS scans in the high sensitivity mode with a mass range of 100–2000 m/z (100 ms accumulation time) of the 20 most intense ions with a 2+ to 5+ charge state. The dynamic exclusion was set to 15 s and the mass tolerance of 50 ppm. A rolling collision energy was selected.

The resulting.wiff files were analysed with MaxQuant [4] version 1.5.2.8 using default settings. The minimal peptide length was set to 7. Trypsin/P was used as the digestion enzyme. Carbamidomethylation of cysteine was set as a fixed modification, and oxidation of methionine and acetyl (protein N terminus) as variable modifications. Up to two missed cleavages were allowed. The mass tolerance for the precursor was set to 0.07 and 0.006 Da for the first and the main searches respectively, and 50 ppm for the fragment ions. TOF recalibration was selected. The files were searched against the *D. melanogaster* UniProt fasta database (July 2014, 41,773 sequences) in which the Biognosys iRT peptide sequences (11 entries) were added. A false discovery rate of 1% was set for peptide and protein levels.

The library was built using Spectronaut 8 from Biognosys [5], using the default settings, from the resulting combined file from the MaxQuant analysis. The library contains 40963 peptides corresponding to 5348 protein groups. The output files from the MaxQuant analysis have been deposited with the ProteomeXchange Consortium via the PRIDE partner repository with the dataset identifier PRIDE: PXD004753.

### 2.4 SWATH data acquisition

Similar LC gradient and mass spectrometer settings as for the IDA acquisition described above were used, but the mass spectrometer was operated in SWATH mode using the following parameters: a 100-ms survey scan was followed by 40 fragment ion spectra from 40 precursor isolation windows. The precursor isolation windows overlap by 1 m/z and thus cover a range of 400–1250 m/z. Precursor isolation windows were as follows: 399.5–422.0, 421.0–441.2, 440.2–456.6, 455.6–471.5, 470.5–484.7, 483.7–497.3, 496.3–510.0, 509.0–522.1, 521.1–533.6, 532.6–545.2, 544.2–555.6, 554.6–566.6, 565.6–577.0, 576.0–587.5, 586.5–597.4, 596.4–607.8, 606.8–618.3, 617.3–629.3, 628.3–640.3, 639.3–651.3, 650.3–663.4, 662.4–675.5, 674.5–688.7, 687.7–701.9, 700.9–715.1, 714.1–728.8, 727.8–743.1, 742.1–758.5, 757.5–774.5, 773.5–791.0, 790.0–809.1, 808.1–827.8, 826.8–848.2, 847.2–871.3, 870.3–897.1, 896.1–927.9, 926.9–965.9, 964.9–1015.4, 1014.4–1097.3, 1096.3–1249.7. The MS2 spectra were recorded with an accumulation time of 40 ms and cover 100–2000 m/z.

### 2.5 SWATH analysis

Spectronaut 8 (Biognosys) was used to analyse the SWATH data. Default settings were used except for the retention time prediction type that was set to dynamic iRT with a correction factor for a window of 2. A Q-value of 10^−5^ (corresponding to a FDR of 0.001% at the peptide level) was used. Proteins with at least two peptides were used for quantitative analysis. The sum of peptide intensities was used as protein intensity. Using this analysis workflow, the median coefficient of variation between the biological replicates was between 10 and 20% for the different time points (Fig. 1B). The fold changes were calculated by comparing the intensities of the proteins in each time point to the 0–4.5 h time point. A volcano-plot was generated using Microsoft Excel to visualize the results (Fig. 1C).

### 2.6 Data availability

All the mass spectrometry data have been deposited with the ProteomeXchange [6] Consortium via the PRIDE partner repository with the dataset identifier PRIDE: PXD003178.

The results from the SWATH analysis of the time course experiment are provided as a Supplementary Table.
