# Supplementary material for: SWATH-MS data of Drosophila melanogaster proteome dynamics during embryogenesis
Source: Data Brief. 2016 Oct 24;9:771–5. doi: 10.1016/j.dib.2016.10.009 (PMC5097952; doi:10.1016/j.dib.2016.10.009)
Supplement: Supplementary file 1 — Supplementary material [file mmc1.docx]

The authors have declared no conﬂict of interest.
